# The dark side of the force: Multiplicity issues in network meta‐analysis and how to address them

**DOI:** 10.1002/jrsm.1377

**Published:** 2019-10-14

**Authors:** Orestis Efthimiou, Ian R. White

**Affiliations:** ^1^ Institute of Social and Preventive Medicine University of Bern Bern Switzerland; ^2^ MRC Clinical Trials Unit, Institute of Clinical Trials and Methodology University College London London UK

**Keywords:** multiple treatments, hierarchical modelling, multiple testing, mixed treatment comparison

## Abstract

Standard models for network meta‐analysis simultaneously estimate multiple relative treatment effects. In practice, after estimation, these multiple estimates usually pass through a formal or informal selection procedure, eg, when researchers draw conclusions about the effects of the best performing treatment in the network. In this paper, we present theoretical arguments as well as results from simulations to illustrate how such practices might lead to exaggerated and overconfident statements regarding relative treatment effects. We discuss how the issue can be addressed via multilevel Bayesian modelling, where treatment effects are modelled exchangeably, and hence estimates are shrunk away from large values. We present a set of alternative models for network meta‐analysis, and we show in simulations that in several scenarios, such models perform better than the usual network meta‐analysis model.

**Highlights**

What is already known?

- Network meta‐analysis (NMA) is a method for synthesizing evidence from multiple studies that compare a range of different interventions for the same disease. NMA simultaneously estimates many relative treatment effects, ie, among all competing interventions in the network.

What is new?

- We argue that the standard NMA model might lead to exaggerated treatment effects regarding the best ranking treatment. It might also be associated with large type I error rates, ie, it can identify differences between treatments which are in truth equally effective or safe.
- In this paper, we present a range of Bayesian, NMA models that account for possible similarities between the treatments by modelling exchangeable treatment effects.
- We show in theory and in simulations that in several scenarios our models can have a much better performance than the usual NMA model.

Potential impact for RSM readers outside the authors' field

- Null hypothesis significance testing should be avoided in NMA.
- When using NMA to synthesize the evidence regarding a clinical question, researchers can use our Bayesian models to take into account similarities between the treatments and possibly obtain better estimates of relative treatment effects.

## 1 INTRODUCTION

Network meta‐analysis (NMA) is a statistical tool for synthesizing evidence from multiple studies comparing a range of alternative treatment options for the same disease.^1^, ^2^, ^3^, ^4^ NMA offers several distinct advantages over a series of standard (pairwise) meta‐analyses, such as an increase in precision and power, the opportunity to compare interventions that have not been compared directly in any studies, and the capacity to provide a ranking of all competing treatments. NMA has been increasingly popular, with hundreds of application being published every year.^5^, ^6^

Despite the popularity of NMA, there have been some concerns regarding the validity of NMA findings. For example, Del Re et al^7^ claimed that considerations related to multiple testing shed doubts on the validity of results of a previously published NMA by Cipriani et al.^8^ This was a NMA on the efficacy of antidepressant drugs, which found several important differences between the drugs. Del Re et al claimed that such findings might be due to multiple testing. They performed simulations where they replicated the network of antidepressants by Cipriani et al, assuming, however, no treatment effects between the drugs. Their simulations showed that 72% of the simulated datasets found at least one statistically significant treatment comparison between the treatments, even though in truth there was none. Thus, they concluded that there is a high chance that the findings of Cipriani et al are false positives and that there might be no actual differences between the drugs. Faltinsen and colleagues^9^ argue that a consensus is needed on how to address multiple testing issues in NMA, and they call for more methodological research regarding the optimal strategy for addressing this issue in future reviews.

The arguments by Del Re et al are based on the concept of statistical significance. It should come as no surprise that using the null hypothesis testing framework in NMA leads to problems, given that the NMA model simultaneously estimates multiple relative treatment effects, ie, comparing all treatments in the network. For example, in a network of 20 treatments, the model estimates 190 different treatment comparisons.

The concept of statistical significance, however, has attracted a lot of criticism lately.^10^, ^11^ For example, as discussed by Gelman and colleagues,^12^, ^13^ it is rather unlikely that any relative treatment effect between different interventions would be *exactly* zero in the real world (although they might be *practically* zero). In that sense, testing the null hypothesis of zero treatment effects using a fixed threshold for the p‐value actually answers a question for which we already know the answer. Thus, retiring statistical significance^11^ would solve the multiple testing issue in NMA, since we would not care anymore about Type I errors.

On the other hand, moving away from a hypothesis testing framework would not fix all issues related to having multiple comparisons in NMA. Problems are expected to arise whenever there is some form of selection procedure performed upon the many estimated treatment effects. This is the case when researchers highlight the most extreme results from a NMA, or when they try to identify the best performing treatment in the network and quantify its effects versus a reference treatment. The latter is to the best of our knowledge common practice, ie, most NMAs are performed with exactly this aim in mind. To illustrate why this might be problematic, let us forget about NMA, and imagine a setting where someone simultaneously estimates 100 different parameters (eg, about the effects of different nutrients to some health outcome) and uses these estimates to draw some conclusion, eg, by highlighting in a guideline the nutrient with the largest effect. Irrespective of whether statistical significance is used to dichotomize results, just by chance some of these 100 estimates will be exaggerated, as compared with their true values. The selection procedure (picking up the largest effect and including it in the guideline) is expected to introduce bias and lead to overstated claims. This is expected to happen even if each specific estimate on each own is unbiassed. To draw parallels, when researchers use the results of a NMA to identify the best performing treatment, they run the exact same risk as in the nutrients case. In other words, and irrespective of whether we choose a p‐value threshold to call our findings “statistical significant,” the fact remains: when we independently estimate a large number of parameters (as the standard NMA model does), we increase the probability of getting some extreme results that do not reflect reality. Such extreme results can then easily be overemphasized in publications and subsequently impact clinical practice, leading to possibly worse patient outcomes and/or unnecessary increase in costs. This raises serious concerns regarding the possible clinical implications. For example, the NMA by Cipriani et al is one of the most highly cited NMAs ever published, and the findings of this paper might have guided clinical practice that affected the lives of millions of patients with depression around the world. Leucht et al placed NMA on the top of the evidence hierarchy when developing treatment guidelines,^14^ and influential organizations such as the WHO have recently adopted NMA when drafting guidelines.^15^ A clear and definite answer as to whether the scientific community can trust findings from NMAs is urgently needed.

In this work, we try to answer this question. We start by discussing the problem theoretically in Section 3. We then review some of the most widely used methods for addressing multiple testing. Then, we propose a series of new NMA models by slightly modifying the standard NMA model. Our models are Bayesian models that incorporate prior information about similarities between the treatments, without requiring strongly informative priors. Such models have previously appeared in the literature,^16^, ^17^, ^18^, ^19^ but have not been discussed in the context of multiplicity. We argue that such models can naturally address the multiplicity issues of the standard NMA model. These models do not employ any form of correction for multiple testing, eg, like Bonferroni. Instead they rely on shrinking effect sizes towards a common mean. In Section 4, we describe a series of simulations that we performed in order to illustrate the multiplicity issues of the standard NMA model, and to compare its performance to our models. In Section 5, we present the application of this new model in two real clinical examples.

## 2 EXAMPLE DATASETS

### 2.1 A network of antidepressants

**Figure 1:**
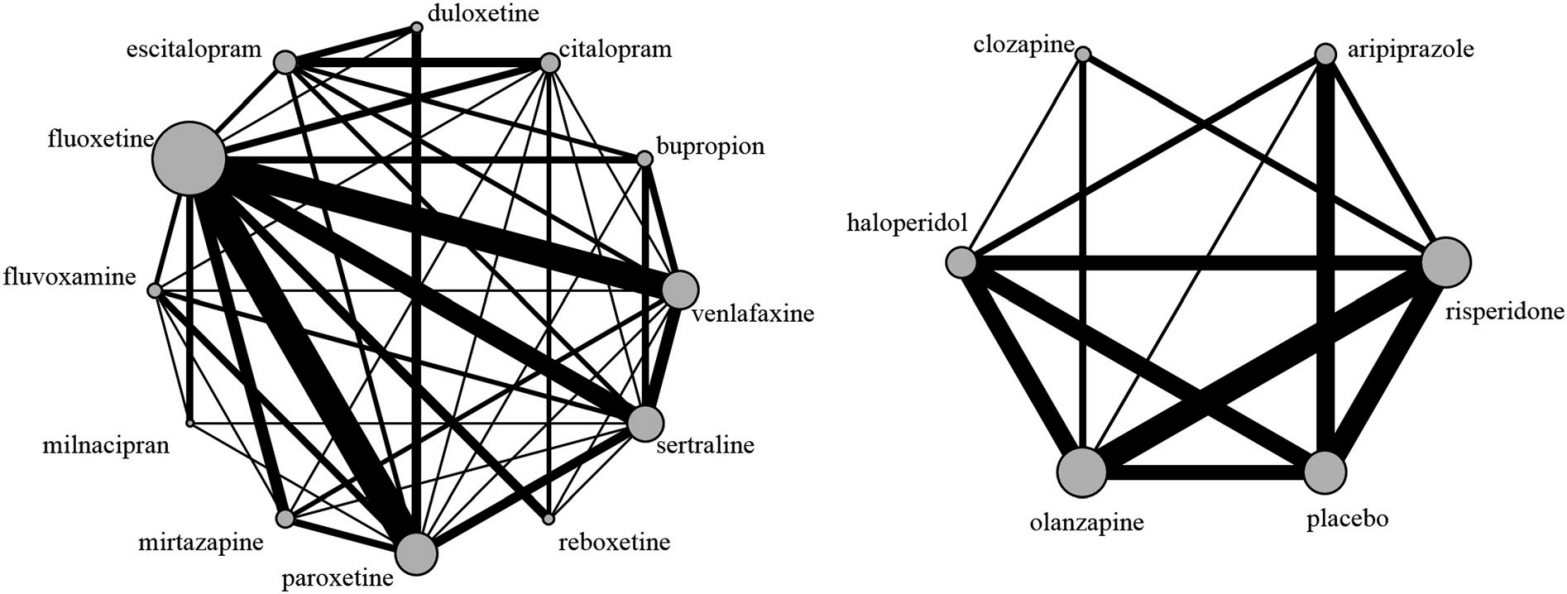
Network of antidepressants (left panel) and neuroleptic drugs (right panel)

Our first example comes from a published NMA by Cipriani et al,^8^ on antidepressant drugs for treating unipolar major depression in adults. The network comprised 111 studies comparing 12 different active drugs (new‐generation antipsychotics). The primary outcome was response to the treatment. This was defined as the proportion of patients who had a reduction of at least 50% from their baselines score on the Hamilton depression rating scale (HDRS), or Montgomery‐Åsberg depression rating scale (MADRS), or who scored much improved or very much improved on the clinical global impression rating scale (CGI) at 8 weeks. The original NMA found important differences between several of the drugs in the network. The reason we chose this particular example is the already discussed concerns raised by Del Re et al .^7^ Here, we re‐analysed this network using the conventional NMA model as well as our models, aiming to highlight the differences between the two approaches, but also to help settle the difference between Cipriani et al and Del Re et al. The network is depicted in the left panel of Figure 1.

### 2.2 A network of neuroleptic drugs for schizophrenia

Our second example comes from a published network comparing five neuroleptic drugs and placebo for patients with schizophrenia.^20^ The outcome we will use is binary efficacy, defined by the authors as “*experiencing* ≥ *20%‐30% reduction from baseline score on the Positive and Negative Syndrome Scale/Brief Psychiatric Rating Scale or a Clinical Global Impression of much improved.*” Thirty studies were included in the dataset. The network is depicted in the right panel of Figure 1.

## 3 METHODS

In this section, we begin by describing the standard NMA model and discuss the multiplicity issue. We then present some approaches that have been used in the past for addressing such issues in other contexts. Subsequently, we propose a way to address the multiple testing issue in NMA, via modelling exchangeable treatment effects.

### 3.1 Multiplicity issues in the standard NMA model

Usual approaches to fitting a NMA^1^, ^21^, ^22^ are based on estimating a set of parameters (the “basic parameters” of the model). These basic parameters are usually set to be the relative treatment effects of each treatment vs a reference treatment. The reference treatment can be chosen arbitrarily, and the other relative effects in the network (ie, between nonreference treatments) can be expressed as linear combinations of the basic parameters. If the network contains *T* treatments, there will be *T* − 1 basic parameters. In total, *T*(*T* − 1)/2 different treatment effects can be estimated from the network.

Let us assume that the dataset comprises *S* two‐armed studies, where each study *i* compares treatments *t*_1*i*_ and *t*_2*i*_. Let us also assume that each study reports the observed relative treatment effect *y*_*i*_ and the corresponding standard error *s*_*i*_. The standard common‐effect (“fixed‐effect”) NMA model can be written as $y_{i}∼N\left(d_{t_{2i}}-d_{t_{1i}},s_{i}^{2}\right)$. *d*_1_ is set to zero without loss of generality, in which case *d*_*t*_ is the effect of treatment *t* vs treatment 1. The *T* − 1 different *d*_*t*_ are the basic parameters of the model. In a Bayesian setting, we usually assign “vague” (flat) prior distributions to the basic parameters, eg, *d*_*t*_∼*N*(0,100^2^), for *t* = 2,3,…,*T*. A random effects NMA model which assumes consistency can be written as follows:

*Model I: Standard NMA model*

$$\begin{array}{l} y_{i}∼N\left(\theta_{i},s_{i}^{2}\right)\\ \theta_{i}∼N\left(d_{t_{2i}}-d_{t_{1i}},\tau^{2}\right)\\ d_{1}=0\\ d_{t\neq 1},\tau ∼\ldots \left(\text{prior distributions}\right) \end{array}$$

Note that including in model I studies that compare more than two treatments requires changing the corresponding univariate normal distributions to multivariate ones. The model above applies to any type of outcome (continuous, binary, and time‐to‐event) and follows a two‐stage, contrast‐based approach. In this approach, at the first stage we obtain study‐specific estimates of relative treatment effects, and at the second stage we perform the NMA. The model can be modified to accommodate a one‐stage analysis, using arm‐based likelihoods; eg, for binary outcomes we could use a binomial likelihood for each arm in each study. Here, *τ* denotes the heterogeneity of treatment effects. Model I assumes a common *τ* for all comparisons in the network.^23^ This is a very usual assumption in NMA, but there are more general formulations.^24^ Setting *τ* = 0 leads to a common‐effect NMA model.

If we use model I to make inference about a prespecified treatment comparison of interest, each estimated treatment comparison is unbiassed when seen in isolation, and there are no multiplicity issues. The problems arise when someone uses a collection of estimates to perform some form of selection.

To explore multiplicity, we consider the case of a star network (ie, when all studies are two‐armed and compare a treatment to the reference) where all relative treatment effects are zero. If we focus on the case where *τ* is known (eg, the common‐effect model *τ* = 0), the *T* − 1 estimates of the basic parameters are independent. Thus, if we use the concept of statistical significance to categorize results, we expect that on average a fraction 1 − (1 − 0.05)^*T* − 1^ of such NMAs will find at least one basic parameter to be statistically significant at the usual 5% level. In networks with many treatments, this false positive rate becomes very large. Eg, if we have a network of *T* = 20 equally effective treatments, there is a 62% chance of at least one statistically significant finding for the basic parameters. The expected fraction of NMAs with at least one statistically significant finding across all treatment comparison in the network (ie, not limiting to basic parameters) will of course be even larger. In summary, if the null hypothesis of no treatment effects in the network holds, a NMA is expected to have a very large false positive rate—the more the treatments in the network, the larger this rate. This implies that if model I is used in conjunction with a statistical significance threshold to answer the question “*is any of the treatments in the network effective/safe?*”, or the question “*are there any differences between the treatments in the network?*”, there is a high probability of spurious findings, if treatments have the same effects.

Moving away from the case of star networks, for the case of networks where nonreference treatments are also directly compared, the basic parameters are no longer independently estimated. Eg, a BC study will jointly inform the basic parameters AB and AC. Thus, the false positive rate for the basic parameters is no longer straightforward to calculate, and it will depend on the geometry of the network. Similarly, extending the model to a random‐effects setting (where *τ* is estimated) makes the theoretical calculation of the expected false positive rate nontrivial, because it will depend on the assumed variance‐covariance structure of the random effects, the number of studies per comparison, and the structure of the network.

Even if we abstain from categorizing findings according to their p‐values, multiplicity can still be an issue. If *T* is large, we expect that by chance alone some of the basic parameters will receive large positive values, and some will receive large negative values. The larger the *T* and the smaller the precision of the studies, the more extreme such results are expected to be. Thus, when identifying the best and worst performing treatment in the network we run the risk of overestimating the corresponding treatment effects and ending up with an exaggerated estimate regarding how well/badly the best/worst treatment performs. This is expected to be the case regardless of what measure we use to identify the best treatment (eg, SUCRAs^25^ or any other measure).

The criticism of Del Re et al^7^ is closely related to what we have discussed so far in this section. One should note, however, that the probability

$$P_{1}=P\left(NMA\;\text{shows treatment effects}\;are\;\text{not zero}\;\right|\;\text{treatment effects}\;are\;\text{zer}\text{o})$$

is not the same as the probability

$$P_{2}=P\left(\text{treatment effects}\;\mathrm{are}\;\text{zero}\;\right|\;\mathrm{NMA}\;\text{shows treatment effects}\;\mathrm{are}\;\text{not zero}).$$

The simulations of Del Re et al and the calculations presented in this section correspond to *P*_1_, but among the two, the clinically interesting quantity is *P*_2_. An attempt to distinguish between *P*_1_ and *P*_2_ naturally leads into a Bayesian way of thinking, since *P*_2_ is a probability only in the Bayesian sense. Following this simple argument, in the next paragraphs we discuss how multiplicity can be naturally addressed within a Bayesian framework.

### 3.2 Currently available methods for correcting for multiple testing

One popular way to fix the multiple testing issue in medical research is to use some form of correction method. Probably, the most popular correction method is the so‐called Bonferroni correction (but there are other similar approaches, eg, see^26^). Let us assume that we want to test *n* independent hypotheses at a desired significance level *a* (where usually *a* = 0.05). Using the Bonferroni method, we instead test each individual hypothesis on a “corrected” level equal to *a*/*n*. This very simple method comes however with several drawbacks. Perhaps, the most important one is that such corrections mitigate the false positive rate at the expense of statistical power. In other words, the Bonferroni as well as other alternative methods focus on holding the rate of type I error (incorrect rejection of the null hypothesis) at the nominal level, but by doing so they increase the rate of type II error (failure to reject an incorrect null hypothesis). Depending on the problem, this increase might be quite important. For these reasons (among others), the use of correction methods to attack the multiple testing issue has been strongly criticized.^27^, ^28^ For the case of NMA where we estimate *T* − 1 basic parameters, following the Bonferroni correction, we would call a basic parameter statistically significant only if the corresponding *P* value was lower than *a*/(*T* − 1). This, however, would lead to a dramatic loss of our power to detect any treatment effects, especially for larger networks. Moreover, it would lead to the counterintuitive case where the interpretation of a result (eg, regarding the comparison of treatments A and B) would depend on seemingly irrelevant pieces of information, such as the number of total comparisons in the network. Eg, the interpretation of results for A vs B would depend on whether A was also compared with D in other, independent trials (while it would not depend on the actual results of these A vs D trials). For these reasons, we believe that the use of Bonferroni (or any other similar) adjustment in NMA would create more problems than it would potentially solve.

Another approach to handle the multiple testing issue is to instead focus on the “false discovery rate”.^29^ This approach does not focus on reducing the familywise error rate (ie, the probability of having at least one false positive) but controls the proportion of false positives. This is a less conservative method than the Bonferroni correction, but it has a larger power. As Gelman et al note,^12^ such methods are generally useful in fields like genetics, but they are expected to be less useful in areas such as NMA, where we usually do not test for thousands of different hypotheses and where true effects are less likely to be exactly zero.

In the next paragraph we describe how the introduction of a small modification to the usual NMA model can mitigate the multiplicity issues while bypassing some of the problems of the aforementioned correction methods. This approach closely follows a Bayesian multilevel modelling approach, discussed by Gelman et al.^12^ In what follows, we start by outlining this approach and adapt it to the case of NMA. Note that multilevel hierarchical models that model exchangeable treatment effects have previously appeared in the NMA literature, eg, by Dakin et al,^16^ DelGiovane et al,^17^ Warren et al,^18^ Owen et al,^19^ and Senn et al^30^ but have not been discussed in relation to multiplicity issues.

### 3.3 Network meta‐analysis models with exchangeable treatment effects

In their paper, Gelman and colleagues^12^ employ data from the “Infant Health and Development Program,” an intervention targeted at premature and low‐birthweight infants. The intervention was administered at eight different sites, and the aim of the analysis was to estimate treatment effects for each site individually. As the authors discuss, the analysis can be performed in two extreme ways. One extreme is what they term “extreme pooling,” which assumes equal (common) treatment effects across sites. This corresponds to the standard, “identical parameters” analysis, eg, as described by Spiegelhalter et al,^31^ page 92. Extreme pooling avoids the multiple testing issue but of course fails to answer the question at hand, ie, it cannot estimate the treatment effect for each individual setting. The other extreme, which Gelman et al term “no pooling,” is to estimate treatment effects in each site separately (ie, independently). Spiegelhalter et al term this “independent parameter” analysis.^31^ This analysis can answer the question of interest, but might be problematic due to multiplicity issues. Gelman et al then describe a middle ground approach which they term “partial pooling,” and which involves random effects modelling. Following this method, the true treatment effects in the sites are assumed to be exchangeable, ie, to be realizations of a common underlying distribution of treatment effects. Instead of inflating the uncertainty of each estimate, this approach shifts the point estimates towards the common mean, effects are shrunk away from extreme values, and the multiple comparisons problem gets resolved without completely diminishing the statistical power. This is the “exchangeable parameters” analysis in Spiegelhalter et al.^31^

We now discuss how these ideas can be applied in a NMA framework. Following the identical treatment effects approach (extreme pooling), we can assume that the effects of all treatments in the network vs the reference are the same, and we can perform a simple pairwise meta‐analysis. This meta‐analysis does not suffer from issues related to multiplicity and will answer the question “*are treatments on average better than the reference?*”. But, it cannot answer the question “*how do different treatments compare to each other?*”. On the other extreme (ie, no pooling), we have the usual NMA model. This analysis tries to estimate relative effects between all treatments, but, as we discussed, it involves estimating a (possibly large) number of parameters, with all the (previously described) problems that this might entail. In order to follow a partial pooling (exchangeable parameters) approach, we propose a set of alternative NMA models.

More specifically, we describe a set of Bayesian models that utilize prior information regarding possible similarities between the treatments. Let us first focus on the usual scenario of a network comprising several active treatments as well as a clearly defined control treatment, such as placebo, no treatment, waiting list, treatment as usual, etc. Moreover, let us assume that the active treatments are somewhat “similar” to each other (but not to control), eg, that they are drugs whose molecules have comparable structure; or that they are treatments known to work through similar biological pathways; or that they are similar surgical operations, etc. In such cases, it might be reasonable to think that the true effects (with respect to a specific effect measure) of these similar treatments form a common underlying distribution of effects. A Bayesian random‐effects NMA model with exchangeable treatment effects for the case when only two‐arm studies are present in the dataset can be written as follows:

*Model II: NMA model with exchangeable treatment effects*

$$\begin{array}{l} y_{i}∼N\left(\theta_{i},s_{i}^{2}\right)\\ \theta_{i}∼N\left(d_{t_{2i}}-d_{t_{1i}},\tau^{2}\right)\\ d_{1}=0\\ d_{k}∼N\left(\mu_{d},\tau_{d}^{2}\right)\;\text{for}\;k=2,3,\ldots ,T\\ \mu_{d},\tau_{d},\tau ∼\ldots \left(\text{prior distributions}\right) \end{array}$$

Weakly informative priors can be employed for *μ*_*d*_ and $\tau_{d}^{2}$. Here we have assumed treatment 1 to be the control treatment, ie, to be dissimilar to the rest of the treatments. The basic parameters of the model (ie, the relative treatment effects of all treatments vs treatment 1) are no longer assigned independent prior distributions as in model I, but are modelled exchangeably. This exchangeability encodes our prior information about the similarity of treatments. Model II “pushes” the estimated relative treatment effects of the different active treatments towards their common mean, to an extent determined by parameter *τ*_*d*_, where *τ*_*d*_ = 0 corresponds to extreme pooling, while *τ*_*d*_ → ∞ corresponds to no pooling. As discussed by Gelman et al,^12^ this model will tend to correct the multiple comparison issue. Moreover, this model makes intuitive sense: when treatments are similar, it is reasonable to assume that their relative effects vs the control follow from a common underlying (normal) distribution, whose parameters we are estimating from the data. When there is large uncertainty regarding the effects of a treatment (eg, when there are only a few small trials that have studied it), then the corresponding estimates of all treatments vs the reference will be strongly pulled towards the overall mean. When uncertainty is small, the corresponding estimate will be shrunk only a little. In general, when we have a densely connected network with many studies per treatment comparison, we expect to find small differences between models I and II. Conversely, in sparse networks with few studies per comparison, differences might be more pronounced.

The only difference between model II and the standard Bayesian NMA model I regards the priors for *d*_*k*_. The standard model assigns independent, flat prior distributions to the effects of the various treatments. Thus, for example, in the standard model, an odds ratio (OR) equal to 0.2 regarding treatment *X* vs control, has the same prior likelihood as an OR equal to 5. In contrast, in Model II, the prior distributions of the effect sizes of the similar treatments vs the control are exchangeable, not independent. This implies, for example, that if the ORs vs the control for all treatments except *X* are found to be centred on 0.2, then we expect the OR of *X* vs the control to be also around 0.2. Thus, we would assign a much higher prior probability to this OR being around 0.2, rather than it being around 5. Note however that the marginal (unconditional) prior for this OR remains flat.

When there are not enough treatments in the network to allow for a precise estimation of the hyperparameters *μ*_*d*_ and *τ*_*d*_, the estimated treatment effects using model II will tend to become similar to the ones estimated using model I. For the extreme case of *T* = 2 treatments, models I and II will become equivalent when it comes to estimating treatment effects, as long as flat prior distributions are used for all parameters in both models. In that case they both correspond to a simple, pairwise meta‐analysis model. Thus, meta‐analysts can safely use model II even for the case of small networks.

Let us now also consider the scenario where there is no clear control treatment in the network. This is the case for the network of antidepressants, which we described in Section 2.1. In such cases, model II is not well suited for the analysis, because it assumes different priors between comparisons vs the reference, and comparisons which are not vs the reference. This is problematic when there is no clear choice for the reference treatment. In such scenarios, we can implement a symmetric version of model II as follows:

*Model III: Symmetric NMA model with exchangeable treatment effects*

$$\begin{array}{l} y_{i}∼N\left(\theta_{i},s_{i}^{2}\right)\\ \theta_{i}∼N\left(d_{t_{2i}}-d_{\left(t_{1i}\right)},\tau^{2}\right)\\ d_{k}∼N\left(\mu_{d},\tau_{d}^{2}\right)\;\text{for}\;\mathrm{all}\;\text{treatments},k\in \left(1,2,3,\ldots ,T\right)\\ \text{corr}\left(d_{k},d_{k'}\right)=0.5\;\;\text{for}\;k\neq k'\\ \mu_{d},\tau_{d},\tau ∼\ldots \left(\text{prior distributions}\right) \end{array}$$

Model III can be more compactly written as ***d*** = (*d*_1_, *d*_2_, …, *d*_*T*_)^'^∼*N*(***μ***, ***Σ***_***d***_), where ***μ =*** (*μ*_*d*_, *μ*_*d*_, …, *μ*_*d*_)^'^ is a vector with *T* identical elements, and ***Σ***_***d***_ is a *T* × *T* matrix with $\tau_{d}^{2}$ in the diagonal and $0.5\tau_{d}^{2}$ in all nondiagonal elements. Note that in this model *μ*_*d*_ does not contribute to the likelihood and can be set to zero. Also note that, as one would intuitively expect, the joint likelihood of treatment effects in model III is identical to the likelihood of the effects between similar treatments in model II (ie, after removing the reference treatment 1), with the only difference being a rescaling of $\tau_{d}^{2}$.

Model II can be extended to the case when active treatments (ie, not the control) belong to multiple classes,^16^, ^18^, ^19^ where *C*_1_ is the first class of similar treatments, *C*_2_ the second class, and so on. For example, in a NMA of interventions for pain relief, one could include placebo, several nonopioid analgesics (class *C*_1_), and several opioid analgesics (class *C*_2_). Similarly, a NMA could include behavioural interventions (*C*_1_) as well as pharmacological interventions (*C*_2_) for the same condition. In such cases, model II can be modified to:

*Model IV: NMA model with exchangeable treatment effects for multiple classes*

$$\begin{array}{l} y_{i}∼N\left(\theta_{i},s_{i}^{2}\right)\\ \theta_{i}∼N\left(d_{t_{2i}}-d_{t_{1i}},\tau^{2}\right)\\ d_{1}=0\\ d_{k}∼N\left(\mu_{d,x},\tau_{d,x}^{2}\right)\;\text{for}\;\mathrm{all}\;\text{treatments}\;k\in C_{x}\\ \mu_{d,x},\tau_{d,x},\tau ∼\ldots \left(\text{prior distributions}\right) \end{array}$$

In this model, we group the basic parameters that correspond to the treatments of each class, and we assign them a common prior distribution. Thus, each class *C*_*x*_ is characterized by a class effect vs the reference (*μ*_*d*,*x*_) and a class‐specific variance ( $\tau_{d,x}^{2}$), which measures the variability of treatment effects within this class. An additional assumption that can be used to simplify model IV is to set *τ*_*d*,*x*_ to be common across all classes, ie, *τ*_*d*,*x*_ = *τ*_*d*_ ∀ *x.* 16, 17, 18, 19 Also, *μ*_*d*,*x*_ could be modelled to be exchangeable across (some of the) classes. Note that model IV can be used if a class includes a few, or even a single treatment. Of course, in this case, the corresponding *μ*_*d*,*x*_ and *τ*_*d*,*x*_ will not be easy to estimate with precision, unless we employ some modelling assumption like the ones discussed above. If all classes in model IV include a single treatment and *μ*_*d*,*x*_ are given independent, flat prior distributions, model IV is equivalent to the standard NMA model, when it comes to estimating *d*_*k*_ and *τ*^2^.

Finally, we can extend our symmetric model III for the case when treatments belong to multiple classes and there is no clear control treatment. Let us choose, without loss of generality, treatment 1 belonging to class *C*_1_ to be the reference treatment of the network. Our symmetric multiclass model can be written as

*Model V: Symmetric NMA model with exchangeable treatment effects for multiple classes*

$$\begin{array}{l} y_{i}∼N\left(\theta_{i},s_{i}^{2}\right)\\ \theta_{i}∼N\left(d_{t_{2i}}-d_{t_{1i}},\tau^{2}\right)\\ d_{k}∼N\left(\mu_{d,1},\tau_{d,1}^{2}\right)\;\text{for}\;\mathrm{all}\;\text{treatments}\;k\in C_{1}\\ \text{corr}\left(d_{k},d_{k^{'}}\right)=0.5\;\;\text{for}\;\mathrm{all}\;\text{treatments}\;k\neq k^{'}\in C_{1}\\ d_{k}∼N\left(\mu_{d,x},\tau_{d,x}^{2}\right)\;\text{for}\;\mathrm{all}\;\text{treatments}\;k\in C_{x}\neq C_{1}\\ \mu_{d,1},\tau_{d,1},\mu_{d,x},\tau_{d,x},\tau ∼\ldots \left(\text{prior distributions}\right) \end{array}$$

where again we could simplify by assuming *τ*_*d*,*x*_ to be common, or by setting *μ*_*d*,*x*_ to be exchangeable among (some of the) classes. This model could be used for example in a NMA of several opioid and nonopioid analgesics, where no placebo‐controlled studies are included.

### 3.4 Considerations regarding statistical power

Because models II‐V incorporate prior beliefs about similarities between some of the treatments, they are expected to have less power to detect differences between them. In contrast, they are expected to have more power to detect differences between treatments not assumed to be similar. Eg, using model II instead of I, we may have less power to detect differences between drugs, but more power to detect differences between drugs and placebo. Using model III, we expect to have less power in detecting treatment effects between any of the treatments in the network. Using models IV and V, we expect to have less power to detect differences between treatments within each class, but more power to detect differences between treatments belonging to different classes. In Section 4, we use simulations to explore how models II and III compares to the standard NMA model I, with respect to the trade‐off between the probability of spurious findings and power.

## 4 SIMULATIONS

### 4.1 Overview

In this section, we describe the simulations we performed to illustrate the multiple testing issue in the standard NMA model (model I), and to compare this model with our proposed models II, III, IV, and V. The primary focus was in comparing the models under different sets of treatment effects, but we also varied other factors, such as network geometry and the existence of random effects.

**Figure 2:**
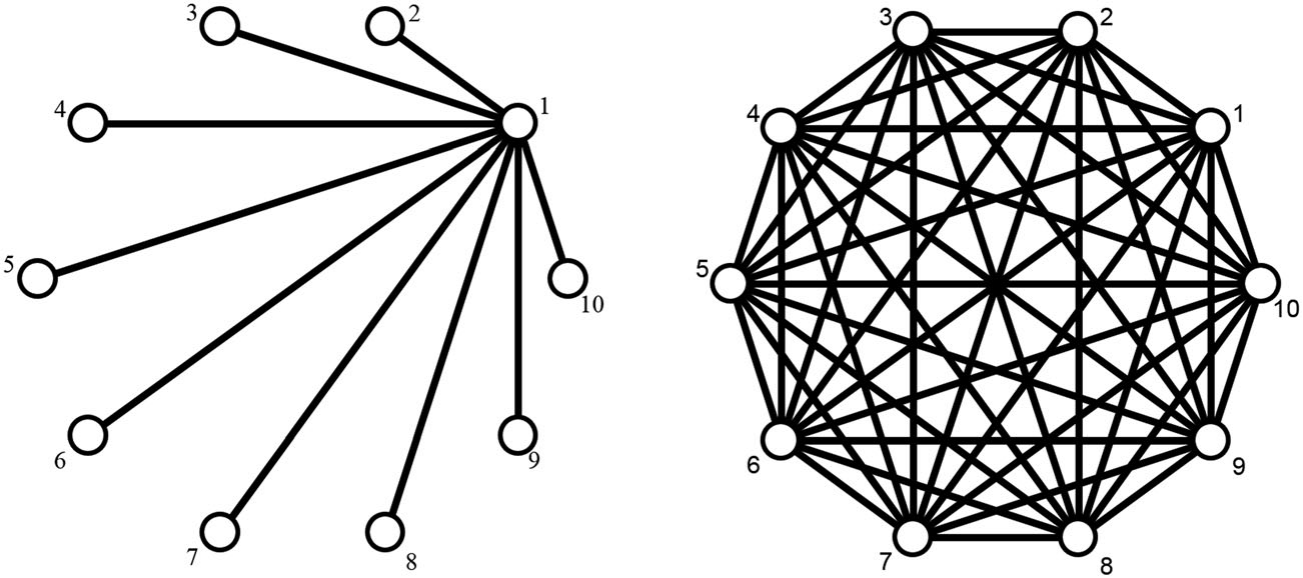
Network structures explored in simulation studies. Star network (left) and fully connected network (right)

We performed two simulation studies, each one including several scenarios. In simulation study 1, we explored the situation when all treatments were equally effective. The initial scenarios had one trial per comparison, and hence heterogeneity was ignored in the analysis. To explore the impact of heterogeneity, we also generated scenarios with multiple trials per comparison. For each scenario except scenario D (where we had three treatments in the network), we explored networks of two contrasting geometries: star networks and fully connected networks (Figure 2). Especially for scenario D, we only generated fully connected networks. We compared models II and III with three variants of model I, using two different priors on the treatment contrasts (flat and informative priors).

In simulation study 2, we explored the situation where some treatments had different effectiveness. We explored several scenarios for the treatment effects, for both star and fully connected networks of 10 treatments. In this study, we simulated only one trial per comparison, ie, heterogeneity was ignored. For the analysis with model I, we used only flat priors. In this simulation study, we also used models IV and V. For model IV, we assumed two classes of treatments: treatments 2 to 5 comprised the first class of similar treatments, and treatments 6 to 10 the second; treatment 1 was the reference. For model V, we assumed two classes, treatments 1 to 5 and 6 to 10.

### 4.2 Data generating mechanisms for each scenario

For each scenario, we simulated 1000 independent datasets. We only simulated two‐armed studies. For each study *i*, we simulated an observed relative treatment effect (*y*_*i*_) and a corresponding standard error (*s*_*i*_). The study‐specific observed effects (*y*_*i*_) were generated in different ways depending on the scenario. For each study, *s*_*i*_ was generated using a chi‐square distribution with one degree of freedom, ie, $s_{i}∼0.5+0.2\;\chi_{1}^{2}$. For scenarios B, we simulated random effects. There we assumed a common variance of the random effects (*τ*^2^) for all treatment comparisons in the network, and we generated it by drawing from a log‐normal distribution *τ*^2^∼*LN*(−2.56,1.74^2^) for each dataset. This was based on the empirical distributions of heterogeneity proposed by Turner et al.^32^ Note that we did not use this distribution for the analyses (see next paragraph). All data were simulated in R.^33^ Codes are available online in https://github.com/esm-ispm-unibe-ch-REPRODUCIBLE/the_dark_side_of_the_force. We investigated eight scenarios (noted as A‐H in Table 1). For each scenario X (where X denotes all scenarios A to H, except C), we simulated data for a star network (termed scenario X.1 in Table 1) and a fully connected network (X.2).

**Table 1:** Overview of the data‐generating mechanisms for all scenarios we explored in our simulations

Name	Description of Simulated Data	Type of Network	Random Effects	Studies per Comparison	Treatment Effect in Study *i*
SIMULATION STUDY 1: zero treatment effects					
A.1	10 treatments. No treatment effects.	Star	✘	1	$y_{i}∼N\left(0,s_{i}^{2}\right)$
A.2		Fully connected			
B.1	10 treatments. No treatment effects.	Star	✓	3	$y_{i}∼N\left(\theta_{i},SE_{i}^{2}\right)$, *θ*_*i*_∼*N*(0, *τ*^2^)
B.2		Fully connected			
C	2 treatments. No treatment effects.	–	✘	5	$y_{i}∼N\left(0,s_{i}^{2}\right)$
D	3 treatments. No treatment effects.	Fully connected	✘	1	$y_{i}∼N\left(0,s_{i}^{2}\right)$
SIMULATION STUDY 2: nonzero treatment effects					
E.1	10 treatments. Equal effects of all active treatments vs a control (treatment 1).	Star (vs treatment 1)	✘	1	$y_{i}∼N\left(\psi_{i},s_{i}^{2}\right)$ *ψ*_*i*_ = 0 for active vs active *ψ*_*i*_ = 1 active vs control
E.2		Fully connected			
F.1	10 treatments. Treatments 1‐5 were worthless treatments (group 1). Treatments 6‐10 were effective treatments (group 2).	Star (vs treatment 1)	✘	1	$y_{i}∼N\left(\psi_{i},s_{i}^{2}\right)$ *ψ*_*i*_ = 0 if both treatments in study were in group 1 or both in group 2 *ψ*_*i*_ = 1 if one treatment in group 1 and the other in group 2
F.2		Fully connected			
G.1	10 treatments. All treatment effects in the network are nonzero.	Star (vs treatment 1)	✘	1	For study *i*, comparing treatments *t*_1*i*_ vs *t*_2*i*_ $y_{i}∼N\left(\psi_{t_{2\iota }}-\psi_{t_{1\iota }},s_{i}^{2}\right)$ where *ψ*_*t*_ = 0 if *t* = 1, and *ψ*_*t*_ = 0.9+0.1 × *t* if *t* > 1
G.2		Fully connected			
H.1	10 treatments. 2 classes of treatments, 2‐5 (class 1) and 6‐10 (class 2). Treatments within each class had equal effects vs treatment 1. Treatment effects vs treatment 1 were set equal to 1(2) for class 1(2), respectively.	Star (vs treatment 1)	✘	1	For study *i*, comparing treatments *t*_1*i*_ vs *t*_2*i*_ $y_{i}∼N\left(\psi_{t_{2i}}-\psi_{t_{1i}},s_{i}^{2}\right)$ where *ψ*_*t*_ = 0 if *t* = 1; *ψ*_*t*_ = 1 if *t* in class 1; *ψ*_*t*_ = 2 if *t* in class 2.
H.2		Fully connected			

Simulation study 1 assumed zero treatment effects in the network and included scenarios A, B, C and D. In scenarios A, we simulated a network of 10 treatments, one study per comparison, giving a total of nine studies for scenario A.1 and 45 studies for scenario A.2. In scenarios B, we assumed 10 treatments, and we simulated three studies per comparison, assuming heterogeneity. In scenarios C, we only had two competing treatments, and each dataset included five studies. In scenarios D, we assumed three treatments in the network, one study per comparison. In simulation study 2 (scenarios E‐H), we assumed nonzero treatment effects in the network. We only used networks of 10 treatments. In scenarios E, we assumed one of the treatments to be the control, and the relative effects of all other treatments vs the control were assumed equal. In scenarios F, we split the treatments in two groups (treatments 1‐5 and 6‐10). Treatments in the same group were assumed to be equally effective, and we assumed different effectiveness between groups. In scenarios G, all treatments had different effectiveness. In scenarios H, we assumed two groups of treatments (treatments 2‐5 and 6‐10) and a control treatment (treatment 1). Treatments in the same group had equal efficacies vs the control. The details of the data generating mechanisms in each scenario are presented in Table 1.

### 4.3 Methods of analysis

For simulation study 1, in each simulated dataset, we fitted model I, ie, the standard Bayesian NMA model with flat (uninformative) priors *d*_*k*_∼*N*(0,100^2^) for all *k* ≠ 1. We repeated the analysis with the same model, using informative priors *d*_*k*_∼*N*(0,1). This was to check whether reasonably informative priors can mitigate issues related to multiplicity without having to resort to more complicated modelling. Next, we analysed each dataset with models II and III, using vague priors for the extra parameters, ie, *μ*_*d*_∼*N*(0,10) and *τ*_*d*_∼*U*(0,2). For scenarios B and C, where we simulated multiple studies per comparison, we used the random‐effects version of these models, with a vague prior for the standard deviation of random effects, *τ*∼*U*(0,5). For the other scenarios, we used the common‐effect version of all models. For simulation study 2, in each simulated dataset, we fitted model I with flat priors, *d*_*k*_∼*N*(0,100^2^). We then repeated the analyses with the common‐effect versions of models II and III, assuming vague priors, *μ*_*d*_∼*N*(0,10) and *τ*_*d*_∼*U*(0,2).

### 4.4 Fitting details

We fitted all models in R using the package rjags. For each model, we fitted two independent chains, 15 000 iterations per chain, with a burn‐in period 5000 iterations. In a sensitivity analysis, to ensure our results were robust, we also used the netmeta 34 command in R^33^ to fit the standard NMA model in a frequentist setting. The code we used for model fitting is also available online in https://github.com/esm-ispm-unibe-ch-REPRODUCIBLE/the_dark_side_of_the_force.

### 4.5 Measures of model performance

After performing the described analyses in each of the 1000 datasets, we extracted the posterior median estimate, the corresponding standard deviation, and the 95% credible intervals (CrI) for all treatment effects. In each analysis, we also identified the best and worst treatment based on the SUCRA values for ranking treatments.^25^

Based on the extracted information, for each scenario of simulation study 1 (where treatments were equally effective) and for each method of analysis, we calculated (a) the mean absolute error for the basic parameters (treatment effects vs treatment 1); (b) the mean bias of the basic parameters; (c) the mean estimate and mean standard deviation for the best vs the worst treatment in the network; (d) the mean estimate and mean standard deviation for the best treatment in the network vs the reference; (e) the percent of datasets that showed with confidence that there were nonzero effects in the network, where in truth there were none. For (e), we checked whether the 95% CrI of the estimated relative treatment effects in the network (among any two treatments) included zero. For scenarios of simulation study 2 (where we assumed nonzero treatment effects in the network) and for each method of analysis, we calculated (e) as the percent of datasets that showed with confidence that there were nonzero effects among treatments that were in truth equally effective, and we additionally calculated (f) power, ie, the percentage of treatment effects that were shown with confidence to be nonzero (95% CrI excluding zero), when in truth they were nonzero.

### 4.6 Results from the simulations

#### 4.6.1 Main findings

A summary of the findings from simulation 1 is shown in Table 2. In scenarios A and B, where we assumed the 10 treatments in the network to be equally effective, models II and III clearly outperformed the standard NMA model I. Model I was associated with higher mean absolute errors; it gave more exaggerated treatment effects regarding the best vs worst treatment, and best vs reference; it also had the largest percentage of datasets showing with confidence that there are nonzero treatment effects among some of the treatments in the network. The results from scenario C showed the equivalence of model I with flat priors and model II, when *T* = 2. In scenario D, where we had a network of only three equally effective treatments, model III clearly outperformed model I. All models were unbiassed in all scenarios A to D.

**Table 2:** Overview of results for simulation study 1 (zero true treatment effects in the network). For all scenarios except B.1 and B.2 we used the fixed effects version of all models

#	Model	Mean Absolute Error (Basic Parameters)	Mean Bias (Basic Parameters)	Mean Estimate For Best Vs Worst Treatment ± SD	Mean Estimate Best Treatment In the Network Vs Reference ± SD	% of NMAs Showing with Confidence Nonzero Treatment Effects
A.1	Model I (flat priors)	0.57	0.01	2.16 ± 1.12	1.10 **±** 0.77	61.9%
	Model I (informative priors)	0.36	0.00	1.30 ± 0.78	0.66 **±** 0.55	35.1%
	Model II	0.24	0.01	0.63 ± 0.58	0.32 **±** 0.37	8.2%
	Model III	0.16	0.01	0.45 ± 0.51	0.23 **±** 0.33	2.4%
A.2	Model I (flat priors)	0.22	−0.02	0.59 ± 0.29	0.28 ± 0.25	61.6%
	Model I (informative priors)	0.19	−0.01	0.56 ± 0.28	0.27 ± 0.24	55.8%
	Model II	0.17	−0.01	0.28 ± 0.23	0.13 ± 0.16	9.0%
	Model III	0.07	0.00	0.17 ± 0.19	0.08 ± 0.14	2.3%
B.1	Model I (flat priors)	0.37	−0.01	1.32 ± 0.74	0.65 ± 0.52	42.6%
	Model I (informative priors)	0.28	−0.01	1.01 ± 0.61	0.49 ± 0.43	32.9%
	Model II	0.17	−0.01	0.40 ± 0.39	0.19 ± 0.23	4.3%
	Model III	0.11	0.00	0.29 ± 0.36	0.14 ± 0.22	0.7%
B.2	Model I (flat priors)	0.17	−0.01	0.43 ± 0.21	0.22 ± 0.18	62.6%
	Model I (informative priors)	0.15	0.00	0.41 ± 0.20	0.20 ± 0.18	60.2%
	Model II	0.13	0.00	0.20 ± 0.17	0.10 ± 0.12	11.0%
	Model III	0.05	0.00	0.13 ± 0.14	0.07 ± 0.11	1.9%
C	Model I (flat priors)	0.23	−0.02	0.23 ± 0.28	–	5.8%
	Model I (informative priors)	0.21	−0.02	0.21 ± 0.27	–	4.5%
	Model II	0.22	−0.02	0.22 ± 0.28	–	5.5%
	Model III	0.16	−0.02	0.16 ± 0.24	–	0.0%
D	Model I (flat priors)	0.48	0.00	0.45 ± 0.54	0.23 ± 0.27	14.5%
	Model I (informative priors)	0.32	0.00	0.32 ± 0.46	0.16 ± 0.23	8.5%
	Model II	0.40	0.00	0.40 ± 0.51	0.20 ± 0.25	10.0%
	Model III	0.24	0.00	0.24 ± 0.40	0.12 ± 0.20	2.3%

A summary of the findings from simulation 1 is shown in Table 3. In scenarios E, where we assumed all treatment effects vs the reference to be equal, model II performed overall the best. The standard NMA model (model I) gave the most exaggerated estimates regarding the best treatment and had the highest rate of false positive findings.

**Table 3:** Overview of results for simulation study 2 (nonzero true treatment effects in the network)

#	Model	Mean Absolute Error (Basic Parameters)	Mean Bias (Basic Parameters)	Best vs Worst Treatment		Best vs Reference Treatment		% of NMAs Showing with Confidence Nonzero Treatment Effects Among Equally Effective Treatments	Power
				Mean Estimate ± SD	True Value	Mean Estimate ± SD	True Value		
E.1	Model I	0.57	0.01	2.37 ± 0.92	1.00	2.14 ± 0.77	1.00	58.9%	36.7%
	Model II	0.24	0.01	1.32 ± 0.45		1.32 ± 0.45		3.0%	69.7%
	Model III	0.32	−0.11	1.37 ± 0.52		1.37 ± 0.52		13.0%	46.4%
	Model IV	0.32	0.00	1.55 ± 0.56		1.53 ± 0.54		17.0%	54.0%
	Model V	0.35	−0.08	1.50 ± 0.56		1.49 ± 0.55		54.8%	22.5%
E.2	Model I	0.22	−0.02	1.28 ± 0.28	1.00	1.28 ± 0.28	1.00	55.6%	93.2%
	Model II	0.17	−0.02	1.08 ± 0.24		1.08 ± 0.24		2.4%	98.8%
	Model III	0.32	−0.27	0.92 ± 0.30		0.92 ± 0.30		28.5%	76.1%
	Model IV	0.19	−0.03	1.14 ± 0.56		1.14 ± 0.56		17.3%	97.3%
	Model V	0.24	−0.15	1.06 ± 0.28		1.06 ± 0.28		28.9%	87.7%
F.1	Model I	0.68	0.01	2.69 ± 1.04	1.00	1.90 ± 0.74	1.00	51.5%	22.7%
	Model II	0.46	0.00	1.30 ± 0.62		1.13 ± 0.51		26.4%	11.7%
	Model III	0.39	−0.08	1.20 ± 0.62		1.04 ± 0.51		14.8%	9.2%
	Model IV	0.47	0.00	1.68 ± 0.69		1.33 ± 0.51		11.3%	31.2%
	Model V	0.43	−0.01	1.53 ± 0.63		1.30 ± 0.51		2.9%	36.6%
F.2	Model I	0.60	0.00	1.46 ± 0.28	1.00	1.15 ± 0.28	1.00	48.0%	94.0%
	Model II	0.57	0.01	1.32 ± 0.28		1.09 ± 0.27		39.9%	92.4%
	Model III	0.21	−0.06	1.28 ± 0.28		1.10 ± 0.25		37.6%	92.2%
	Model IV	0.58	0.00	1.25 ± 0.24		1.10 ± 0.22		12.8%	99.0%
	Model V	0.55	0.00	1.20 ± 0.22		1.15 ± 0.28		5.7%	99.5%
G.1	Model I	0.61	0.01	2.73 ± 0.84	1.80	2.61 ± 0.76	1.80	–	16.5%
	Model II	0.32	0.00	1.79 ± 0.47		1.79 ± 0.47		–	17.9%
	Model III	0.36	−0.10	1.93 ± 0.54		1.92 ± 0.53		–	16.0%
	Model IV	0.38	0.00	2.00 ± 0.53		1.99 ± 0.53		–	18.0%
	Model V	0.42	−0.08	2.00 ± 0.54		1.99 ± 0.54		–	18.7%
G.2	Model I	0.30	−0.01	1.88 ± 0.28	1.80	1.88 ± 0.28	1.80	–	40.5%
	Model II	0.27	0.01	1.74 ± 0.27		1.74 ± 0.27		–	31.5%
	Model III	0.26	−0.17	1.67 ± 0.29		1.67 ± 0.29		–	38.0%
	Model IV	0.27	−0.03	1.74 ± 0.26		1.74 ± 0.26		–	43.0%
	Model V	0.31	−0.11	1.67 ± 0.29		1.67 ± 0.29		–	43.1%
H.1	Model I	0.68	0.01	3.02 ± 0.82	2.00	2.90 ± 0.74	2.00	46.5%	32.4%
	Model II	0.47	0.00	2.13 ± 0.51		2.13 ± 0.51		8.5%	29.3%
	Model III	0.41	−0.09	2.25 ± 0.55		2.25 ± 0.54		17.8%	29.5%
	Model IV	0.47	−0.01	2.33 ± 0.51		2.32 ± 0.51		5.7%	41.6%
	Model V	0.51	−0.08	2.34 ± 0.52		2.32 ± 0.51		7.5%	44.9%
H.2	Model I	0.40	−0.02	2.21 ± 0.28	2.00	2.21 ± 0.28	2.00	43.0%	95.0%
	Model II	0.38	−0.02	2.13 ± 0.28		2.13 ± 0.28		35.2%	93.9%
	Model III	0.24	−0.12	2.06 ± 0.28		2.06 ± 0.28		38.6%	94.2%
	Model IV	0.37	−0.03	2.07 ± 0.25		2.07 ± 0.25		6.5%	99.2%
	Model V	0.42	−0.15	1.96 ± 0.26		1.96 ± 0.26		16.7%	97.2%

In scenarios F, where treatments were split in two groups, models V performed best. Model I had the worst performance in terms of exaggerating the effects of the best treatment, and also false positive findings. Models II and III had the smallest power.

In scenario G.1, where all treatment effects in the network were nonzero and the network was star‐like, model I was the worst in terms of mean absolute error and exaggeration of treatment effects, and second worst in power. Model II had the best performance overall, followed by IV. In scenario G.2, all models performed comparably, but model II had the lowest, and model IV the largest power.

In scenarios H, model IV had overall the best performance. Model I had the largest mean absolute error, it showed the most exaggerated results regarding the effects of the best treatment and had the largest false positive rate.

In summary, in almost all cases, the standard NMA model (model I) led to the most exaggerated claims regarding treatment effects of the best treatment in the network. This exaggeration was especially pronounced for star networks, rather than fully connected ones. In scenarios, where no treatment effects were simulated, model I gave the largest proportion of networks that found nonzero relative treatment effects. In the next sections, we provide some additional results for some of the scenarios.

#### 4.6.2 Reversal of effects

For scenarios G, where all treatments were assumed to be differently effective, we calculated the percent of estimates for which the 95% CrI did not include zero, but showing a wrong direction of effects (eg, when treatment 2 was found to be worse than treatment 1, although in truth it was better). For scenario G.1, using model I, we found that 1.1% of all estimated treatment effects across all datasets showed a reversed effects. For all other models, this was below 0.00%. Similarly, for G.2, the percentages were 0.03%, 0.00%, 0.02%, 0.00%, and 0.06% for models I to IV, respectively. These results highlight another possible advantage of modelling treatment effects exchangeably.

#### 4.6.3 Treatment rankings

In all scenarios of simulation study 1, due to the way we generated the data in this scenario treatments were identical. Thus, we would ideally like to see on average small differences between the maximum and minimum SUCRA value in each NMA. This, however, was not the case for model I. For example, in Scenario A.1, the mean value for the maximum SUCRA was 0.84 (ranging from 0.63 to 1.00), while for the minimum SUCRA 0.16 (from 0.00 to 0.39). Using model I with informative priors instead, SUCRA values were largely unaffected. Conversely, with model II, max (SUCRA) was 0.72 (0.56 to 0.97) and min (SUCRA) was 0.27 (0.02 to 0.46); with model III, max (SUCRA) was 0.67 (0.54 to 0.95), and min (SUCRA) was 0.32 (0.05 to 0.47).

#### 4.6.4 Further findings

Aiming to show the equivalence between model I with flat priors and model II in scenario C, we calculated the mean absolute difference of the estimated treatment effects across all datasets between these two models, and we found it to be 0.002. The mean absolute difference of the corresponding standard deviations was 0.001. These results confirm what we theoretically anticipate, ie, models I and II are equivalent when *T* = 2.

## 5 APPLICATION TO REAL DATASETS

### 5.1 Antidepressants

We used the data from Cipriani et al^8^ to fit the usual random effects NMA model I. Following the original publication, we chose fluoxetine to be the reference treatment for the model. We also fitted the symmetric random‐effects NMA model III. We opted for the symmetric model because there was no placebo or other obvious control treatment in the network. We fitted the models in R, using the rjags package. For both models, we used *τ*∼*U*(0,3) as a prior for the standard deviation of random effects. For model III, we assumed *μ*_*d*_∼*N*(0,1) and *τ*_*d*_∼*N*(0,1)*I*(0, ), ie, the positive part of *N*(0,1). We used two independent chains per analysis, and we performed 50 000 iterations per chain, discarding the first 15 000.

In both models, the posterior median for *τ* was 0.11 [95% CrI 0.01; 0.20]. For model III, *τ*_*d*_ was estimated as 0.23 [0.12; 0.44]. All results regarding relative treatment effects are shown in Table 4. As expected, model III pulled estimates towards the null, ie, it was more conservative. Taking for example mirtazapine vs reboxetine, model I estimated an odds ratio (OR) 2.04 [1.52; 2.78], while model III gave 1.61 [1.19; 2.22]. Similarly, for fluvoxamine vs mirtazapine, the standard model estimated 0.71 [0.55; 0.93], while the new model 0.78 [0.61; 0.97]. The ranking of the treatments based on the SUCRA values was very similar in the two models and is shown in Table 5.

**Table 4:** Results for the antidepressants network. All relative treatment effects are OR for response (95% credible intervals). Lower triangle: estimates from the usual NMA model I. OR > 1 favours the column‐defining treatment. Upper triangle: estimates from NMA model III. OR > 1 favours the row‐defining treatment

Bupropion	0.96[0.79; 1.18]	1.05[0.84; 1.33]	0.83[0.69; 1.00]	1.05[0.90; 1.25]	1.05[0.84; 1.35]	1.02[0.79; 1.33]	0.83[0.66; 1.02]	1.04[0.87; 1.27]	1.32[1.01; 1.79]	0.88[0.74; 1.05]	0.86[0.72; 1.01]
0.98[0.79; 1.23]	Citalopram	1.10[0.88; 1.37]	0.86[0.73; 1.02]	1.10[0.93; 1.28]	1.10[0.88; 1.39]	1.05[0.82; 1.37]	0.85[0.69; 1.04]	1.09[0.91; 1.28]	1.37[1.08; 1.82]	0.91[0.75; 1.09]	0.89[0.74; 1.06]
1.10[0.84; 1.43]	1.11[0.87; 1.43]	Duloxetine	0.79[0.65; 0.95]	1.00[0.82; 1.22]	1.00[0.78; 1.28]	0.96[0.74; 1.27]	0.78[0.61; 0.98]	0.99[0.82; 1.19]	1.25[0.95; 1.69]	0.83[0.67; 1.03]	0.81[0.65; 1.00]
0.82[0.67; 1.00]	0.83[0.70; 0.99]	0.75[0.61; 0.92]	Escitalopram	1.27[1.10; 1.47]	1.27[1.02; 1.61]	1.22[0.96; 1.59]	0.99[0.81; 1.20]	1.25[1.08; 1.47]	1.59[1.20; 2.13]	1.05[0.89; 1.25]	1.03[0.88; 1.20]
1.09[0.91; 1.30]	1.10[0.93; 1.32]	1.00[0.79; 1.23]	1.33[1.12; 1.56]	Fluoxetine	1.00[0.82; 1.23]	0.96[0.77; 1.20]	0.78[0.65; 0.93]	0.99[0.87; 1.12]	1.25[0.99; 1.61]	0.83[0.72; 0.96]	0.81[0.71; 0.93]
1.11[0.83; 1.45]	1.12[0.87; 1.45]	1.01[0.75; 1.37]	1.35[1.04; 1.75]	1.02[0.81; 1.28]	Fluvoxamine	0.96[0.73; 1.27]	0.78[0.61; 0.97]	0.99[0.80; 1.20]	1.25[0.95; 1.69]	0.83[0.66; 1.03]	0.81[0.65; 1.00]
1.06[0.77; 1.49]	1.08[0.79; 1.52]	0.97[0.69; 1.41]	1.3[0.94; 1.82]	0.98[0.74; 1.32]	0.96[0.69; 1.37]	Milnacipran	0.81[0.61; 1.05]	1.03[0.81; 1.28]	1.30[0.98; 1.79]	0.86[0.67; 1.11]	0.85[0.65; 1.08]
0.79[0.62; 1.01]	0.80[0.63; 1.01]	0.72[0.54; 0.94]	0.96[0.76; 1.20]	0.72[0.6; 0.88]	0.71[0.55; 0.93]	0.74[0.53; 1.02]	Mirtazapine	1.27[1.05; 1.54]	1.61[1.19; 2.22]	1.06[0.88; 1.30]	1.04[0.87; 1.25]
1.06[0.87; 1.30]	1.09[0.90; 1.30]	0.98[0.79; 1.20]	1.30[1.10; 1.52]	0.98[0.85; 1.12]	0.96[0.76; 1.22]	1.00[0.74; 1.33]	1.35[1.11; 1.64]	Paroxetine	1.27[1.00; 1.67]	0.84[0.71; 0.99]	0.82[0.70; 0.96]
1.61[1.20; 2.17]	1.64[1.25; 2.13]	1.47[1.06; 2.04]	1.96[1.49; 2.63]	1.49[1.16; 1.89]	1.45[1.05; 2.04]	1.52[1.03; 2.17]	2.04[1.52; 2.78]	1.52[1.16; 2.00]	Reboxetine	0.66[0.50; 0.87]	0.65[0.49; 0.85]
0.87[0.71; 1.05]	0.88[0.72; 1.08]	0.79[0.62; 1.01]	1.06[0.88; 1.27]	0.80[0.69; 0.93]	0.79[0.61; 1.01]	0.81[0.59; 1.10]	1.10[0.88; 1.37]	0.81[0.69; 0.96]	0.53[0.41; 0.71]	Sertraline	0.98[0.83; 1.15]
0.85[0.7; 1.01]	0.86[0.71; 1.04]	0.78[0.61; 0.98]	1.04[0.86; 1.23]	0.78[0.68; 0.89]	0.77[0.6; 0.98]	0.80[0.58; 1.08]	1.08[0.88; 1.32]	0.79[0.68; 0.93]	0.52[0.4; 0.69]	0.98[0.83; 1.16]	Venlafaxine

**Table 5:** Ranking of antidepressants using SUCRA values

Drug	Model I	Model III
Mirtazapine	0.91	0.89
Escitalopram	0.87	0.88
Venlafaxine	0.82	0.82
Sertaline	0.77	0.77
Citalopram	0.52	0.54
Bupropion	0.48	0.43
Milnacipran	0.36	0.40
Paroxetine	0.35	0.32
Fluvoxamine	0.31	0.32
Duloxetine	0.31	0.31
Fluoxetine	0.30	0.30
Reboxetine	0.00	0.02

Based on the results of Table 4, we conclude that there is a strong evidence of difference in efficacy between some of the antidepressants. These results are unlikely to be false findings due to multiplicity.

In a series of sensitivity analysis, we (a) fitted the standard NMA model I after choosing a different treatment to be the reference, and with *τ*∼*U*(0,5); (b) fitted model III using less informative prior distributions: *τ*∼*U*(0,5), *μ*_*d*_∼*N*(0,10), and *τ*_*d*_∼*U*(0,2); and (c) fitted the random‐effects version of model II. These sensitivity analyses did not give substantially different results for most comparisons. In practice, one might want to perform additional analyses using models IV and/or V, after splitting the drugs into classes. However, given that the main focus of this paper is on the methods, we did not pursue this any further.

We provide the code and data that we used for model fitting both for R and for OpenBUGS^35^ in https://github.com/esm-ispm-unibe-ch-REPRODUCIBLE/the_dark_side_of_the_force.

### 5.2 Neuroleptic drugs

We used the data by Klemp et al^20^ described in Section 2.2 to first fit the usual random effects NMA model I. We also fitted our NMA model II. We chose model II over the symmetric model III, because in this example there was an obvious control treatment in the network, ie, placebo. We fitted the models the same way as in Section 5.1.

The estimate for *τ* was similar in the two models. In the standard model, it was 0.20 [0.02; 0.41], while in model II it was 0.22 [0.03; 0.42]. In model II, *τ*_*d*_ was estimated 0.09 [0.01; 0.74], and *μ*_*d*_ was 0.87 [0.45; 1.26]. The estimated treatment effects are shown in Table 6. In both models, all drugs were shown to be more efficacious than placebo. However, results from model II pointed to smaller differences between some of the active treatments. For example, the OR for aripiprazole vs clozapine was 0.63 [0.40; 1.00] using the standard model, while using model II it was estimated to be 0.73 [0.47; 1.09]. This highlights that model II might be more conservative in identifying differences between active treatments. The ranking of the treatments was unchanged using the two models and is shown in Table 7.

**Table 6:** Results for the neuroleptic drugs network. All relative treatment effects are ORs for response (and 95% credible intervals). Lower triangle: estimates from the standard NMA model I. OR > 1 favours the column‐defining treatment. Upper triangle: estimates from model II. OR > 1 favours the row‐defining treatment

Aripiprazole	0.73[0.47; 1.09]	1.18[0.86; 1.61]	0.78[0.56; 1.05]	2.13[1.59; 2.86]	0.77[0.56; 1.04]
0.63[0.40; 1.00]	Clozapine	1.64[1.05; 2.44]	1.06[0.78; 1.49]	2.94[2.00; 4.35]	1.05[0.76; 1.49]
1.20[0.83; 1.69]	1.89[1.27; 2.78]	Haloperidol	0.65[0.51; 0.92]	1.79[1.33; 2.50]	0.64[0.49; 0.93]
0.73[0.52; 1.02]	1.15[0.80; 1.67]	0.60[0.48; 0.79]	Olanzapine	2.78[2.04; 3.70]	0.99[0.79; 1.23]
2.08[1.54; 2.86]	3.33[2.17; 5.00]	1.75[1.30; 2.38]	2.86[2.17; 3.85]	Placebo	0.36[0.27; 0.48]
0.72[0.51; 1.00]	1.14[0.78; 1.64]	0.60[0.46; 0.79]	0.99[0.78; 1.23]	0.34[0.26; 0.45]	Risperidone

**Table 7:** Ranking of neuroleptic drugs using SUCRA values

Drug	Model I	Model II
Clozapine	0.90	0.84
Risperidone	0.76	0.77
Olanzapine	0.73	0.75
Aripiprazole	0.39	0.41
Haloperidol	0.23	0.23
Placebo	0.00	0.00

## 6 DISCUSSION

In this paper, we highlighted the possible implications of the multiplicity issue in NMA. We presented the problems associated with multiplicity using both theoretical arguments as well as simulations. We showed that the model commonly used for NMA may give exaggerated estimates of the effects of the treatment identified to be the best in the network. It may also be associated with a high probability of (falsely) showing differences between treatments, when actually there is none.

The problems stem from the fact that the NMA model simultaneously estimates tens (or even hundreds, depending on the network size) of relative treatment effects. By chance alone, some of these estimates may be very large. Any selection procedure following the estimation (eg, identifying the best ranking treatment, or focusing on “statistically significant” results) will lead to exaggerated findings. These issues will be more pronounced when the number of treatments in the network is large.

In this paper, we discussed how a set of alternative Bayesian NMA models can be used to address problems associated with multiplicity. The key difference between these models and the standard NMA approach is that the former model treatment effects exchangeably, ie, assuming that the true effects of the treatments are realizations of one or more common underlying normal distributions. This small modification to the standard NMA model is enough to mitigate the multiplicity issue. The price to pay is a possible decrease in the statistical power to detect differences between treatments assumed to be similar (although there might be an increase in the power to detect differences between nonsimilar treatments).

Simulations showed that in a range of different scenarios, our models had a better performance than the standard model. The latter gave in almost all cases the larger mean absolute error regarding the estimates of the basic parameters; it gave the most exaggerated estimates regarding the relative effects of the best treatment vs the reference, and best vs worst treatment. Also, it was worse in terms of the trade‐off between false positives (falsely identifying differences among equally effective treatments) and power. Eg, in scenario E.1 (star network, all treatments had equal effects vs the reference), when we switched from model I to model II, we saw that the effects of the best treatment vs reference were estimated with a much smaller error, power almost doubled (from 37% to 69%), while false findings reduced from 55% to 3%. Likewise, in scenario G.1, where all treatments were generated to have different effectiveness, exaggeration of effects was much more pronounced in model I, while power was more or less similar across all models.

One limitation of our simulations is that we did not include scenarios with multi‐arm studies. Note however that the problems with multiplicity arise in NMA when some form of selection among the estimated effects is performed, regardless of whether these estimates used data from multi‐arm studies. At the same time, as we saw in simulations, when the network includes a lot of information (eg, when it is more densely connected), the exaggeration of the effect of the best treatment is expected to be smaller. In that sense, keeping the number of studies and number of patients per study‐arm constant, networks with multi‐arm trials are expected to give less exaggerated effects than networks with two‐arm studies only. Also, let us note that the power rates we found for some of the scenarios are unrealistically large. This was an artefact of the (arbitrary) choices for the data generating mechanisms, and the fact that for some scenarios we considered fully connected networks. In practice, it is rather unlikely that a network of 10 treatments will ever be fully connected, and the high power we showed in some scenarios should not be interpreted as a general feature of NMA. In addition, readers should keep in mind that the aim of our simulations was to compare the relative performance of the models, not to assess the absolute power of NMA in usual conditions of data availability.

The practice of dichotomizing evidence according to “statistical significance” has been heavily criticized lately. We also think that researchers should abstain from characterizing evidence as statistically significant or not, and we showed in our simulations that this practice can be especially problematic in NMA, for all models we used. However, as we presented in this article, the multiplicity issue in NMA is not limited to multiple testing. Thus, although dropping the notion of statistical significance is a step in the right direction, it is by itself not enough to safeguard us against all multiplicity issues in NMA. In this paper, we showed how utilizing prior information regarding similarities among treatments can help address the issue and possibly lead to more trustworthy NMAs.

One important aspect of our models is that they require some subjective decisions to be made. The decision of which treatments to include in the network (or how to group treatments in models IV and V) may be more important in our models as compared with standard NMA. Eg, with model II, including in the network an older drug that is less efficacious might lead to more conservative estimates about the newer drugs. In this scenario, however, if the older drug is substantially different from the newer drugs (eg, in terms of the molecule structure), it might make more sense to include it in a separate class, ie, using model IV.

Also, the assumption of exchangeability of treatment effects might be subject to criticism, eg, in cases where drugs use different biological pathways. Furthermore, exchangeability might be a difficult concept to convey to clinicians. One approach might be to ask questions such as

“You are trying to assess treatment X, and you are told that treatment Y is effective/ineffective/safe/unsafe. Would this information about Y impact your judgement about X?”

“You are trying to assess treatment X, and you are told that one of the other treatments in the network is effective/ineffective/safe/unsafe. Would you need to know which other treatment it was?”

Answers “yes” and “no,” respectively, would support an exchangeability assumption. Decisions about exchangeability among treatments need to be taken a priori, by content experts, taking into account existing knowledge about the nature of treatments (and not the results of the studies in the network). Moreover, these decisions should be clearly reported and justified, preferably in the protocol stage of the review. Of course a Bayesian analysis will always include some level of subjectivity, but to our view this is not a disadvantage, as long as all decisions are transparent.

An obvious question at this point is whether one should prefer one of our NMA models for the primary analysis, over the standard NMA model. We think that whenever there is prior knowledge regarding possible similarities among some of the treatments in the network, our models should be considered. The answer might also depend on the nature of the research question. For example, for the case of a NMA of a serious safety outcome (eg, mortality), where several drugs are compared with placebo, researchers might be more interested in increasing the power to detect differences vs placebo, rather than between drugs. In such case, our models might be more appropriate to use.

Another case where our models might be very useful is when for some treatments there is limited and/or biassed evidence. Imagine that there is available only a single study, or a few small studies, of a new drug vs placebo. Assume that in these studies, due to publication bias (or due to other biases, or due to chance alone), the effect of the new drug is grossly exaggerated. This may be a relatively common situation; Fanelli et al showed that earlier studies often show inflated results.^36^ In a case like this, the standard NMA model may identify this drug as being the best in the network and may estimate large treatment effects vs all other drugs. Conversely, our models will give less biassed estimates in this scenario, by pushing the effectiveness of this drug towards the mean effectiveness of the rest of the drugs.

The models we have described could be further extended for the case when we have information about predictors at the level of the treatments. Eg, let us assume that we have a network of similar drugs being compared with placebo, and that there is available information regarding a treatment characteristic that is expected to affect the outcome, such as the duration of the treatment. In that case, instead of pulling the treatment effects towards the overall mean, we could use a multilevel regression model to pull treatment effects towards the regression line. In cases where treatments in the network cannot be assumed similar (so that our hierarchical models cannot be used), we think that the considerations presented in this paper can still be useful in highlighting the potential issues with performing a NMA to identify the best performing treatment and also in using the concept of statistical significance in NMA.

To summarize, we think that the models we proposed are a useful addition to a network meta‐analyst's arsenal of statistical methods, and that in several scenarios they should be preferred over the standard NMA model.

## Data Availability

We provide the code and data that we used for model fitting both for R and for OpenBUGS^35^ in https://github.com/esm-ispm-unibe-ch-REPRODUCIBLE/the_dark_side_of_the_force.
